# Eating disorders and associated mental symptoms in Swedish elite athletes and a comparison group

**DOI:** 10.1007/s44192-026-00547-9

**Published:** 2026-07-13

**Authors:** Frida Ejderstedt, Mathilda Karlsson, Klara Edlund, Madeleine Ahlgren, Haris Pojskic, Anna Katarina Melin

**Affiliations:** 1 School of Psychology, Sport and Health Sciences, University of Portsmouth, Portsmouth, UK; 2 Occupational health, psychology and sports sciences, University of Gävle, Gävle, Sweden; 3 Department of Clinical Neuroscience, Section of Psychology, Karolinska Institutet, Solna, Sweden; 4 Department of Sport Science, Linnaeus University, Kalmar, Växjö, Sweden

**Keywords:** Eating disorder examination interviews, Depression, Exercise addiction, Psychological flexibility, Elite sport

## Abstract

**Aim:**

This cross-sectional two-step study aimed to investigate the prevalence of eating disorder (ED) symptoms and associated mental health difficulties among elite athletes and an age-and sex matched comparison group.

**Methods:**

National team athletes were recruited through national sport federations, while participants in the comparison group were recruited via social media. A total of 408 participants (athletes *n* = 256; comparison group *n* = 152) (72.1% female), completed an anonymous online survey comprising validated psychometric measures of ED symptoms, exercise addiction, depression and psychological flexibility. Participants who screened positive for ED symptoms and providing contact details were invited to undergo a clinical assessment using the ED Examination Interview (EDE 17.0D).

**Results:**

Overall, 25.0% (*n* = 102) reported ED symptoms, with a higher prevalence in the comparison group than among athletes (37.5% vs. 17.6%, *p* < 0.001). Interviews were completed by 78.0% (*n* = 18) of athletes and 47.0% (*n* = 16) of comparison group participants who screened positive for ED symptoms, confirming an ED diagnosis in all but one athlete. Furthermore, 18.6% (*n* = 76) reported clinically relevant symptoms of depression, and 22.8% (*n* = 93) of exercise addiction. Associations were observed between ED symptoms and symptoms of exercise addiction, depression, and psychological inflexibility in the total sample, among athletes and in the comparison group.

**Conclusion:**

ED symptoms were common among athletes and the comparison group and were associated with depressive symptoms, problematic exercise behaviour, and psychological inflexibility. To safeguard athlete health and optimise performance, national sports organisations should implement systematic preventive strategies, promote early identification, establish clear referral pathways, and ensure access to coordinated multidisciplinary care.

## Background

Eating behaviour can be conceptualised as a continuum ranging from health-promoting nutritional and training practices, through disordered eating (DE) characterised by behaviours such as restrictive dieting and body dissatisfaction, to clinically diagnosed eating disorders (EDs) as defined in the Diagnostic and Statistical Manual of Mental Disorders (DSM-5) [1].

Other Specified Feeding or Eating Disorder (OSFED), formerly classified as Eating Disorder Not Otherwise Specified (EDNOS) in DSM-IV, is the most frequently reported ED diagnosis in both the general population and elite athlete cohorts [2–4].

EDs are severe, and multifaceted psychiatric disorder associated with substantial psychiatric comorbidity, including depression, and anxiety [5], as well as increased rates of non-suicidal self-harm [3]. Individuals with EDs also demonstrate markedly heightened mortality rates [6]. Furthermore, depending on the clinical ED manifestation, a broad spectrum of acute and chronic medical complications may occur [5]. Hence, EDs pose substantial emotional, psychological, and economic burdens on affected individuals and society at large [7]. However, also individuals with DE behaviours engage in health-impairing behaviours such as restricted and/or loss of control eating behaviour as well as excessive or compulsive exercise without fully meeting the criteria for a clinical ED diagnosis. In athletic populations, DE behaviours appear to be more common than clinically diagnosed EDs [8–10], and have been identified as an important predictor of sport dropout among young elite athletes [11].

### Prevalence across athletic and non-athletic populations

ED symptoms are highly prevalent worldwide, particularly among females, and epidemiological evidence suggests that the prevalence has approximately doubled from 4% in the early 2000s to around 8% during 2013–2018 [12]. DE behaviours and EDs have commonly been reported as more prevalent among athletes than the general population; however, findings across studies remain inconsistent due to methodological variability and sport-specific differences [13]. Research among Norwegian elite athletes in the early 2000s, using gold standard methods, found that 20% of female and 8% of male national team athletes met criteria for clinically verified EDs, compared with 8% and 0.5% among female and male non-athlete controls, respectively [8]. On the contrary, a recent Swedish study among elite and advanced rock-climbers and non-athletic controls reported no significant group differences in reported symptoms of EDs, although male controls reported nearly three times more ED symptoms than male climbers [14].

### Medical health complications of eating disorders

DE behaviours and EDs are associated with a wide range of acute and chronic medical complications affecting nearly every major organ system [15–18]. These complications arise from mechanisms such as long-term and/or severe energy deficiency, malnutrition at any body weight, compensatory behaviours such as purging, and recurrent binge-eating episodes. Documented clinical outcomes include electrolyte disturbances, reproductive dysfunction, endocrine alterations, gastrointestinal complications, poor bone health, cardiovascular risk factors such as bradycardia, orthostatic hypotension, endothelial dysfunction and dyslipidaemia, as well as neuropsychiatric impairments [19].

Emerging longitudinal evidence shows that DE behaviours predict subsequent performance decrements and interruptions in training and competition [20, 21]. Among female athletes, DE behaviours and EDs are associated with an increased risk of musculoskeletal injuries [22]. ED pathology in sport frequently co-occurs with long-term and/or severe low energy availability and the syndrome Relative Energy Deficiency in Sport (REDs) [1, 23]. REDs may manifest through multiple physiological, psychological, and performance-related impairments and is frequently associated with maladaptive eating and training behaviours, including compulsive exercise and reduced psychological well-being [23, 24]. Although REDs and DE behaviours/EDs share many features, such as endocrine alterations, reproductive disturbances, cardiovascular risk factors and poor bone health, REDs may occur without DE behaviours and EDs and vice versa [1, 23].

### Mental health complications of eating disorders

DE behaviours and EDs are consistently associated with a broad profile of psychological difficulties. Depression is common and frequently co-occurs with EDs, functioning both as a potential antecedent and as an outcome of DE behaviours [25, 26]. Moreover, within high-performance environments, ED symptoms are strongly associated with greater psychological distress, diminished well-being, and substantial barriers to help-seeking [20, 21]. Emerging evidence suggests that psychological inflexibility may represent an important transdiagnostic process underlying both the development and maintenance of ED pathology and associated psychological distress [27]. Psychological flexibility was initially defined within the Acceptance and Commitment Therapy (ACT) framework as the ability to remain open, aware, and adaptive in response to internal experiences such as anxiety, depressive affect, and stress, while engaging in values-driven behaviour [28]. It is further described as the capacity to flexibly navigate adverse or challenging circumstances while maintaining actions aligned with personally meaningful goals and values [27]. In athletic settings, psychological flexibility is considered particularly important, as athletes are repeatedly exposed to stressors, performance-related anxiety and competitive pressure [29, 30].

Participation in sport has traditionally been viewed as beneficial for psychological well-being. However, although moderate physical activity confers mental health benefits, excessively high training frequency (> 23 sessions per month) has been associated with poorer mental health outcomes in both athletes and non-athletes [31]. Moreover, maladaptive exercise behaviours are recognised as contributory factors in the development and maintenance of ED pathology [32]. Dysfunctional exercise behaviours, including exercise dependence and compulsive training behaviours, are frequently observed among individuals with ED pathology [33–35]. Indeed, the reported prevalence of excessive and compulsive exercise in patients with EDs is 48% [36].

Dysfunctional exercise behaviour is best conceptualised as a continuum of maladaptive exercise patterns, ranging from broadly health-impairing activity behaviours often intertwined with eating pathology to more specific constructs such as compulsive exercise and exercise addiction. Exercise addiction is characterised by loss of control, withdrawal symptoms, tolerance, and continued engagement despite adverse consequences [37]. Pathological exercise behaviour is a significant clinical concern among athletes and frequently remains unrecognised within sports medicine settings [38].

### Mental health burden in athletes

The considerable burden of mental health symptoms among elite athletes has become an increasingly important focus within sports medicine and athlete welfare research [10, 39]. Previous research has shown that elite athletes experience mental health symptoms at rates comparable to or exceeding those of the general population [40, 41]. However, recent evidence suggests that former elite athletes have over double the point prevalence of anxiety and depression compared to general population estimates [42].

Various risk factors contribute to mental health challenges in athletes, including sport-specific stressors such as injury, competitive selection pressure, and general risk factors such as adverse life events or maladaptive coping [43]. Psychological inflexibility has been shown to contribute to elevated psychological distress, including anxiety, depressive symptoms and maladaptive emotional responses in sport settings [29, 30]. Athletic injury can adversely affect mental health, while psychological distress can impair performance, creating a bidirectional cycle of vulnerability [44]. Indeed, sports injury has for more than three decades been reported to be a common trigger for EDs in female athletes [45, 46].

### Risk factors for eating disorders in athletes

Athletes are considered at increased risk of EDs [1] and affect both genders, although prevalence remains higher in females, and in sports where leanness is perceived to provide a competitive advantage [8].

Athletes are exposed to several sport-specific risk factors for DE behaviours and EDs, including sports injury ([45]; Arthur-Cameselle et al., 2025), and high training loads [31, 47]. Sociocultural pressures and body dissatisfaction play critical roles in the development of DE behaviours and EDs, especially during adolescence [48, 49]. External and internal weight-pressure for performance [8, 21], and performance-driven restrictive eating and heightened drive for thinness [50], are frequently reported risk factors for EDs among athletes. Furthermore, a prevailing culture of comparison within sport [25], alongside coaching practices that may inadvertently legitimise or reinforce unhealthy weight‑control behaviours, has also been identified as a contributing factor [80]. Besides DE behaviours and EDs, these risk factors may lead to co-occurring, depression, anxiety and sleep disturbance that further intensify this cycle [51, 52].

There is substantial documentation of the co-occurrence of EDs and depression. Attia and[5, 25, 26]. Athlete-focused studies confirm that DE behaviour is significantly associated with both current depressive symptoms and life-time depressive disorders in competitive sport settings [25, 53]. Further, maladaptive exercise is recognised as central symptom in emerging and fully developed EDs pathology, where compulsive training and exercise dependence is frequently observed [32–35]. Emerging evidence also suggests that psychological inflexibility may represent a key underlying mechanism linking ED pathology with its psychological correlates, potentially mediating both vulnerability and maintenance processes within high-performance sport environments [27].

Collectively, DE behaviours and EDs represent complex and debilitating conditions with substantial implications for mental health, physical health, and exercise behaviour. Although growing evidence has documented associations between ED pathology, depression, maladaptive exercise behaviours, and psychological factors in athletes, research examining these relationships simultaneously remains limited. In particular, the role of psychological flexibility in relation to ED symptoms and broader mental health outcomes in elite sport is poorly understood.

Therefore, the primary aim of the present study was to investigate the prevalence of ED symptoms among Swedish elite athletes compared to an age- and sex-matched comparison group. A secondary aim was to examine associations between ED symptoms, depressive symptoms, exercise addiction, and psychological flexibility.

## Materials and methods

This cross-sectional, observational two-step study forms part of the larger REDs Sweden project (ClinicalTrials.gov ID: NCT06371963, 2024-04-12). All procedures followed the Declaration of Helsinki and were approved by the Swedish Ethical Review Authority (No 2021-07031-01). Written informed consent was obtained from all participants before study enrolment.

### Recruitment and data collection

All Swedish sport federations with national teams (*n* = 65) were invited to participate through the Swedish National Federation of Sport, the Swedish Olympic Committee, and social media platforms. Federations were asked to distribute an invitation email containing a link to the study information page and anonymous online survey to all senior athletes competing at national-team, development-team, or professional level (Tier 4 according to the six-tier Participation Classification Framework) [54].

Federation responses varied considerably. Three federations declined participation, 24 did not respond, and five forwarded the invitation to national coaches without further follow-up. Five reminder emails were distributed (three weekly and two fortnightly reminders). Among the 33 federations that agreed to participate, recruitment procedures differed; some selected athletes to invite, others distributed to all eligible national-team athletes, and some shared the study through the federation webpage or social media channels. Consequently, the number of athletes reached, and the survey response rate could not be determined. Furthermore, recruitment through social media enabled some athletes to participate independently of their federation’s involvement.

The comparison group consisted of individuals participating in sport or physical activity at a recreational level (Tier 1) or regularly training ~ 3 times a week with competitive aspirations (Tier 2) according to the Participation Classification Framework [54]. Recruitment was conducted through social media. Individuals currently competing in elite sport were excluded from the comparison group. Inclusion criteria for all participants were age ≥ 18 years and sufficient proficiency in Swedish.

Data were collected between May 2023 and May 2024 using a two-step design comprising: (1) an anonymous online survey completed by all participants; and (2) a semi-structured clinical interview. At the end of the survey participants were invited to provide contact details if they wished to participate in the clinical inteview, which was subsequently administered to a subsample screening positive for ED symptoms.

### Survey instruments

The survey collected demographic information, including self-reported age, sex, height and body weight. Body mass index (BMI) was calculated as weight (kg) divided by height squared (m²). Weight fluctuation was defined as the difference between the participants’ highest and lowest self-reported adult body weight excluding pregnancy-related changes. The following validated psychometric instruments were administered.

### The eating disorder examination questionnaire (EDE-Q 6.0)

The EDE-Q [55] is a 28-item self-report measure derived from the Eating Disorder Examination (EDE) interview [56] and assesses core ED psychopathology during the proceeding 28 days. The instrument comprises four subscales: Restraint, Eating Concern, Shape Concern, and Weight Concern. Items are rated on a 7-point scale ranging from 0 to 6, with higher scores indicating greater psychopathology. Subscale scores are calculated as the mean of constituent items, and the Global score represents the mean of all four subscales. Established cut-offs indicating clinically relevant ED symptoms are ≥ 1.68 for males and ≥ 2.30 for females [57, 58]. The EDE-Q has demonstrated validity in both female and male elite athletes [59].

### Exercise addiction inventory (EAI)

The EAI is a six-item instrument designed to identify individuals at risk of exercise addiction [60]. Responses are recorded on a five-point Likert scale ranging from 1 (strongly disagree) to 5 (strongly agree), generating total scores between 6 and 30. Higher scores indicate greater exercise addiction risk. A cut-off score of ≥ 24 has been recommended in Scandinavian populations [61]. Previous studies support instruments’ construct validity, internal consistency, and single-factor structure [61, 62].

### Major depression inventory (MDI)

The MDI assesses depressive symptoms according to DSM-IV-TR diagnostic criteria [63]. The scale comprises 10 items rated from 0 (at no time) to 5 (all the time), producing total scores ranging from 0 to 50. Higher scores indicate greater depressive symptom severity. In accordance with previous recommendations, a score of ≥ 26 was used to indicate clinically relevant depressive symptoms of at least moderate severity [63].

### Swedish acceptance and action questionnaire (SAAQ)

SAAQ is a six-item measure assessing psychological inflexibility and experiential avoidance [64]. Items are rated on a 7-point Likert scale from 1 (never true) to 7 (always true) providing total scores ranging from 7 to 42. Higher scores reflect greater psychological inflexibility. Previous validation studies have demonstrated acceptable reliability and concurrent validity in Swedish populations [64].

### Eating disorder examination interview (EDE 17.0D)

Participants screening positive for ED symptoms who provided contact information and consented to further participation were invited to complete an online clinical interview using the EDE 17.0D [65]. The EDE is a semi-structured, clinician-administered investigator-based interview and is considered gold standard assessment of ED psychopathology [56]. The interview assesses eating behaviour and ED symptoms during the proceeding 28 days including and contains the same four subscales as the EDE-Q. Most items are rated on a scale from 0 (absence of the feature) to 5 or 6 (extreme severity). Interviews were conducted by two experienced and EDE licenced clinicians (AM, KE). Interview duration typically ranged from 45 to 75 min. The Swedish version (17.0D) was used throughout the study.

### Categorisation of leanness and non-leanness sports

Athletes were categorised according to previous classification systems proposed by Martinsen et al., [50] and Mancine et al., [66]. Sports were classified as leanness-demanding when lower body mass or a specific physique was considered advantageous for performance (e.g., endurance, aesthetic, and weight-category sports), and as non-leanness-demanding when body mass was not a primary determinant of performance (e.g., floorball, badminton, and bandy) [67].

To account for variation within weight-category sports, athletes were additionally classified according to the international competition weight class in which they competed. Athletes competing in lower weight categories (e.g., powerlifting < 63 kg for females and < 79 kg for males) were classified as participating in leanness-demanding disciplines, whereas athletes competing in higher weight categories (e.g., > 84 kg for females and > 120 kg for males) were classified as participating in non-leanness-demanding disciplines. Table 1 displays the full categorisation of included sports.

**Table 1:** The sport disciplines represented in the study, categorised into leanness and non-leanness demanding sports

Leanness demanding sports (*n* = 117)	Non-leanness demanding sports (*n* = 139)
Biathlon, Cheerleading, Climbing, Cross-country skiing, Cycling, Functional fitness, Gymnastics, Judo, Martial Arts, Middle-long distance running, Obstacle course; Orienteering, Powerlifting, Swimming, Triathlon, Weightlifting	Archery, Badminton, Bandy, Bowling, Canoe, Curling, Equestrian, Fencing, Floorball, Football, Golf, Handball, Ice hockey, Motor sport, Powerlifting (+ 84/+120 kg), Rowing, Rugby, Sailing, Tennis, Water Skiing, Weightlifting (+ 86/+110 kg)

### Statistical analysis

Of the 603 survey respondents, 408 participants were included in the final analysis following sex- and age-matching between athletes with participants from the comparison group. All athletes were retained and served as the reference-group. Participants from the comparison group were sequentially excluded until no significant difference in age or sex distribution remained between groups, resulting in the exclusion of 195 comparison group participants. The final sample was therefore considered comparable with respect to age and sex.

The distribution of continuous variables was assessed using the Shapiro–Wilk test. Normally distributed variables are presented as mean ± SD and were compared using independent samples t-tests. Non-normally distributed variables are presented as median [25–75th interquartile range (IQR)] and were compared using Mann–Whitney U tests. Categorical variables are presented as frequencies (n) and percentages (%) and were compared using chi-square tests. Cramer’s V was calculated to assess effect size for categorical comparisons [68].

Associations between EDE-Q Global score and scores on the EAI, MDI, and SAAQ, were examined using Spearman’s rank-order correlation (r_s_). Correlation coefficients were classified as weak (0.10–0.39), moderate (0.40–0.69), or strong (0.70–1.00) [68]. All tests were two-tailed, and statistical significance was set at *p* < 0.05. Data were analysed using IBM SPSS Statistics for Windows, version 30.0 (IBM Corp., Armonk, NY, USA).

## Results

A total of 408 participants were included in the analysis, comprising of 256 athletes and 152 participants in the comparison group. Overall, 72.1% of participants were females. Median age was 24.0 [20.0–28.0] years and median BMI was 22.6 [21.2–24.5] kg/m^2^. Athletes reported significantly more weekly training hours than participants in the comparison group, whereas participants in the comparison group reported lower body mass and greater weight fluctuation (Table 2).

Overall, 25.0% (*n* = 102) of participants screened positive for symptoms of EDs. Among athletes who screened positive, 51.0% agreed to participate in the clinical interview, of whom 78.0% (*n* = 18) completed the assessment. In the comparison group, 60.0% agreed to participate and 47.0% (*n* = 16) completed the interview. A clinical ED diagnosis was confirmed in all interviewed participants, except one elite athlete.

The prevalence of ED symptoms was significantly higher among females than males (29.3% vs. 14.0%, *p* = 0.002), and among participants from the comparison group than athletes (37.5% vs. 17.6%, *p* < 0.001).

Among athletes, 21.7% (*n* = 39) of females and 7.9% (*n* = 6) of males screened positive for ED symptoms. Athletes with ED symptoms had a lower body mass (Table 2) and were more likely to compete in leanness-demanding sports compared with athletes without symptoms (25.6% vs. 10.8%, *p* < 0.001).

Compared with participants in the comparison group, athletes with ED symptoms reported greater weight fluctuation (Table 2), and lower dietary restraint scores (Table 3).

In the comparison group, 41.2% (*n* = 47) of females and 26.3% (*n* = 10) of males screened positive for ED symptoms. Participants in the comparison group with ED symptoms reported more training hours per week and greater weight fluctuation compared with those without ED symptoms (Table 2).

### Mental health symptoms

Overall, 18.6% (*n* = 76) of participants reported clinically relevant depressive symptoms. A significant higher prevenance was observed in the comparison group than among athletes (23.7%, n=35 vs.15.6%, n=40, p=0.049).

Symptoms indicative of exercise addiction were identified in 22.8% (*n* = 93) of participants, including 21.5% of athletes and 25.0% of participants in the comparison group. No significant group difference was observed (*p* = 0.413).

Participants with ED symptoms reported significantly greater symptoms of exercise addiction, more severe depressive symptoms, and higher levels of psychological inflexibility than participants without ED symptoms. These differences were observed in the total sample and within both athletes and the comparison group (Table 4).

Moderate positive correlations were observed between EDE-Q Global scores and symptoms of exercise addiction, depressive symptoms, and psychological inflexibility (Table 5).

**Table 2:** Descriptive and sport characteristics between athletes and the comparison group with and without symptoms of EDs

Characteristics	Athletes			Comparison group		
	All (*n* = 256)	Symptoms of EDs (*n* = 45)	No ED symptoms (*n* = 211)	All (*n* = 152)	Symptoms of EDs (*n* = 57)	No ED symptoms (*n* = 95)
Age (years)	23.0 [2.0–27.0]	22.0 [19.5–24.5]	23.0 [20.0–27.0]	25.0 [21.0–28.0]	24.0 [20.0–28.0]	25.0 [22.0–28.0]
Height (cm)	172 [167.0–180.0]	169.8 ± 8.5^#^	173.0 [167.0–181.0]	169.0 [164.0-177.0]	170 [162.0–177.5]	169.0 [165.0–175.0]
Body mass (kg)	67.5 [60.0–76.0]*	64.0 [58.0–72.0]^#^	70.0 [61.0–78.0]	64.5 [60.0–75.0]	64.0 [60.0–7.0]	65.0 [60.0–73.0]
BMI (kg/m^2^)	23.0 [21.0–24.8]	22.2 [21.1–23.8]	23.1 [21.4–24.7]	22.0 [21.0–24.0]	22.3 [20.3–26.0]	22.5 [21.0– 23.9]
Weight fluctuation (kg)	8.0 [6.0–11.0]*	9.0 [7.0–12.0]^‡^	8.0 [5.0–11.0]	11.5 [7.0–19.8]	7.0 [4.0– 13.0]^¥^	7.0 [3.0–12.0]
Training time (h/week) (*n* = 328)	14.0 [10.0–18.0]*	15.0 [11.0–20.0]^‡^	13.0 [10.0–17.0]	9.7 ± 4.0	11.12 ± 4.0^¥^	8.9 ± 3.8

EDs, eating disorders; BMI, Body Mass Index; Weight fluctuation refers to the highest versus lowest body mass with current height (besides pregnancy). *p < 0.05 significant difference between all athletes and all participants in the comparison group. ‡*p* < 0.05 significant difference between athletes and participants in the comparison group with symptoms of EDs. #*p* < 0.05 significant difference between athletes with and without symptoms of EDs. ¥*p* < 0.05 significant difference between participants in the comparison group with and without symptoms of EDs. Normally distributed data are presented as mean ± SD. Non-normally distributed data are presented as median and 25–75 interquartile range.

**Table 3:** EDE-Q scores between athletes and participants in the comparison group with symptoms of EDs

EDE-Q subscales	All (*n* = 102)		Females with EDs (*n* = 86)		Males with EDs (*n* = 16)	
	Athletes (*n* = 45)	Comparison group (*n* = 57)	Athletes (*n* = 39)	Comparison group (*n* = 47)	Athletes (*n* = 6)	Comparison group (*n* = 10)
Global score	3.1 [2.6–3.8]	3.3 [2.6–4.2]	3.2 [2.6–3.8]	3.6 [2.7–4.4]	2.7 [2.1–3.6]	2.7 [2.3–3.5]
Dietary restrain	2,4 [1.9–3.7]*	3.6 [2.6–4.2]	2.74 ± 1.26^‡^	3.32 ± 1.33	3.2 [1.9–4.1]	3.7 [2.5–4.8]
Eating concerns	2.2 ± 0.9	2.3 ± 1.2	2.4 [1.6–2.8]	2.4 [1.6–3.6]	2.1 [1.6–2.8]	1.4 [0.9–2.8]
Shape concerns	4.3 [3.4–5.0]	4.4 [3.7–5.4]	4.4 [3.6–5.1]	4.6 [3.9–5.4]	2.9 [1.4–4.1]	3.2 [1.2–5.1]
Weight concerns	3.7 ± 1.1	3.9 ± 1.1	3.8 [3.0–4.4]	3.8 [3.4–4.8]	3.2 [2.1–4.1]	3.4 [2.5–4.2]

EDs, eating disorders; EDE-Q, Eating Disorder Examination Questionnaire; *p < 0.01 significant difference between all athletes and all participants in the comparison group with symptoms of EDs: ‡*p* < 0.05 significant difference between female athletes and participants in the comparison group with symptoms of EDs: Normally distributed data are presented as mean ± SD. Non-normally distributed data are presented as median and 25–75 interquartile range.

**Table 4:** Symptoms of mental health problems in participants with and without symptoms of EDs

Mental health surveys	All (*n* = 408)			Athletes (*n* = 256)			Comparison group (*n* = 152)		
	ED symptoms (*n* = 102)	No ED symptoms (*n* = 306)	*p *value	ED symptoms (*n* = 45)	No ED symptoms (*n* = 211)	*p *value	ED symptoms (*n* = 57)	No ED symptoms (*n* = 95)	*p *value
EAI score	23.0 [20.0–25.0]	19.5 [17.0–22.0]	< 0.01	22.8 ± 2.9	20.0 [17–23]	< 0,001	23.0 [20.0–26.0]	18.6 ± 4.3	< 0,001
EA % (n)	41.2 (42)	16.7 (51)	< 0.001	37.8 (17)	18.0 (38)	0.005	43.9 (25)	13.7 (13)	< 0.001
MDI score	25.0 [20.0–29.5]	17.0 [12.0–21.0]	< 0.01	23.8 ± 7.8	17.0 [12.0–21.0]	< 0.001	26.0 ± 8.3	16.0 [12.0–20.0]	< 0.001
Depression % (n)	45.1 (46)	9.8 (30)	< 0,001	40.0 (18)	10.4 (22)	< 0.001	49.1 (28)	8.4 (8)	< 0.001
SAAQ score	21.5 [15.0–27.0]	14.0 [9.0–19.0]	< 0.01	20.1 ± 8.3	14.0 [9.0–19.0]	< 0.001	22.1 ± 8.6	14.0 [9.0–18.0]	< 0.001

EA, Participants at risk of Exercise Addiction; EAI, Exercise Addiction Inventory; Depression, Participants at risk of major depression; MDI, Major Depression Inventory; SAAQ, Swedish Acceptance and Action Questionnaire; Normally distributed data are presented as mean ± SD. Non-normally distributed data are presented as median and 25–75 interquartile range. Significance levels were set to *p* < 0.05.

**Table 5:** Correlations between EDE-Q global score and mental health problems

Mental health questionnaires	All (*n* = 408)	Athletes (*n* = 256)	Comparison group (*n* = 152)
	EDE-Q	EDE-Q	EDE-Q
EDE-Q	1	1	1
EAI	0.40 (0.31–0.48)	0.41 (0.30–0.51)	0.42 (0.28–0.55)
MDI	0.49 (0.41–0.57)	0.42 (0.32–0.53)	0.60 (0.47–0.69)
SAAQ	0.44 (0.36–0.52)	0.38 (0.27–0.49)	0.51 (0.37–0.62)

EDE-Q, Eating Disorder Examination Questionnaire; EAI, Exercise Addiction Inventory; MDI, Major Depression Inventory; SAAQ, Swedish Acceptance and Action Questionnaire; Spearman’s rank correlations (r_s_) with 95% CI. The associations between EDE-Q and all other questionnaire scores had significance levels of *p* < 0,001.

## Discussion

This two-step study examined the prevalence of ED symptoms and their associations with mental health in Swedish elite athletes compared with an age- and sex-matched comparison group. Although the prevalence of ED symptoms was higher in the comparison group, results confirm that ED symptoms are common in elite sport and associated with symptoms of depression, exercise addiction and psychological inflexibility.

### Prevalence of eating disorder symptoms

The prevalence of ED symptoms among athletes in the present study (18%) aligns with the global mean prevalence of 19% reported in a recent systematic review and meta-analysis including more than 70,000 athletes across 177 countries [69]. Similarly, a Swedish longitudinal study among national‑team gymnasts assessed over 12 months found a prevalence of 17% (*n* = 94; 56% female), with no significant temporal change [70]. A recent study among Danish athletes reported 17% prevalence in national‑team athletes (*n* = 410; 51% female), compared with 31% in sub‑elite athletes (*n* = 206; 51% female) competing at high level, but below national team level [25].

However, prevalence estimates differ substantially depending on screening tool and study population characteristics [71]. For example, Lichtenstein et al., [25] reported a prevalence of 14% using the EDE-Q and 22% using the five-item Sick Control, One Stone (6.5 kg), Fat, Food (SCOFF) [72] in Danish athletes, mirroring the higher sensitivity of SCOFF reported in a clinical interview study [59]. The present findings therefore corroborate the literature showing that the prevalence of ED symptoms in elite sport likely falls within the 15–25% range, depending on methodology. Since the sample in the current study included exclusively national team athletes, the prevalence may be lower than in studies also including sub-elite athletes, who have been shown to exhibit higher rates of ED symptoms [25]. Greater access to medical, nutritional, and psychological support at the national-team level may partially explain these differences.

### Sex differences

Consistent with previous research [8, 13, 25, 69, 73], female athletes in the current study displayed a higher prevalence of ED symptoms than male athletes. This difference is well documented and may be driven by factors such as greater body dissatisfaction, a stronger drive for thinness, and higher levels of anxiety among females compared to male athletes [74]. Similar patterns were observed within the comparison group, emphasising an increased vulnerability in women compared to men. However, recent data show rising rates of clinically diagnosed EDs among young male athletes (Sundgot‑Borgen et al., 2025), suggesting that ED symptoms remain under-recognised and under‑diagnosed in males [75]. One reason could be that men and women may display different ED manifestations, for example increased drive for thinness among women, while increased drive for muscularity in men [76], differences that are not adequately captured by commonly used instruments such as the EDE-Q.

### Sport type and risk

In the present study, more athletes in leanness-demanding sports reported ED symptoms than those in non-leanness sports, consistent with robust evidence from multiple countries [8, 77, 78]. The performance-driven emphasis on low body mass and leanness across several sport disciplines remains one of the strongest predictors of ED development.

### Comparison with the comparison group

In the present study the prevalence of ED symptoms was lower among elite athletes than participants in the comparison group which is in line with some recent studies [14, 79]. Although a Meta analysis including 56 studies reported similar prevalence of ED psychopathology between female athletes and controls [78], earlier two-step cross-sectional studies using gold standard methods found higher prevalence among elite athletes than sex- and aged matched controls [8, 45, 80]. However, more recent population-based research indicates that ED symptoms are increasing in the general population [12], especially after the Covid-19 pandemic [2], which may explain the reverse pattern observed in the present study.

In the present study, female participants in the comparison group had higher dietary restraint compared to female athletes, while there was no difference between groups regarding body dissatisfaction or any other ED psychopathology. This contrasts with earlier studies reporting lower levels of body dissatisfaction among athletes compared to nonathletes [78]. This could indicate sport participation and exercise to be a potential protective factor [70, 78], yet body dissatisfaction has been emphasised as a robust predictor of the development of ED psychopathology, in both athletic and non-athletic samples [81].

Notably, the finding that participants in the comparison group demonstrated a higher prevalence of ED symptoms than elite athletes challenge the traditional assumption that competitive sport is invariably associated with increased ED risk. Although caution is warranted due to differences in recruitment procedures and the cross-sectional design, the finding may reflect a broader increase in ED symptoms in the general population, as well as potential protective factors associated with organised elite sport, including structured training environments and access to specialised medical and psychological support.

### Eating disorder symptoms, mental health, and psychological flexibility

The present study found that participants with ED symptoms reported significantly higher levels of depressive symptoms, exercise addiction symptoms, and psychological inflexibility than those without ED symptoms. Furthermore, positive associations were observed between ED symptom severity and measures of depression, exercise addiction and psychological flexibility across both athletes and participants in the comparison group. These findings are consistent with previous athlete-focused research demonstrating that DE behaviours are closely associated with depressive symptoms and broader psychological distress in competitive sport settings [25, 53]. Similarly, evidence from the general population identifies depression as one of the mental health conditions most strongly associated with ED psychopathology [82].

A notable finding was the association between ED symptoms and psychological inflexibility. Psychological inflexibility has increasingly been recognised as a transdiagnostic process underlying a range of psychological disorders and is characterised by rigid behavioural patterns, experimental avoidance, and difficulties adapting behaviour in response to changing circumstances [83, 84]. Emerging evidence suggests that such processes are also closely linked to ED psychopathology and may contribute to both the development and maintenance of maladaptive eating and exercise behaviours [27, 85]. This may be particularly relevant in elite sport, where athletes are routinely exposed to performance pressure, injury, competitive stress, and high personal expectations [29, 30]. In such environments, psychological inflexibility may increase vulnerability to both emotional distress and rigid behavioural strategies related to eating, body weight, and exercise [51]. Although the cross-sectional design precludes causal inference, our findings suggest that psychological inflexibility may represent a shared vulnerability factor contributing to the co-occurrence of ED symptoms, depressive symptoms, and exercise addiction.

Taken together, these findings support the value of integrated screening approaches that assess not only eating-related symptoms but also associated mental health difficulties and underlying psychological processes. Such an approach may facilitate earlier identification of athletes at risk and help inform more comprehensive prevention and intervention strategies.

### Exercise addiction

Consistent with previous research, symptoms of exercise addiction were more common among participants with ED symptoms than among those without ED symptoms [34, 86]. Participants with ED symptoms also reported greater training volumes, further supporting the close relationship between maladaptive exercise behaviour and eating pathology. Notably, the prevalence of exercise addiction observed in both athletes and the comparison group was substantially higher than that reported in previous studies using the same screening instrument [25, 86, 87]. Differences in sample characteristics, recruitment methods, and increasing societal exercise engagement may partly explain this discrepancy.

### Clinical implications

The high prevalence of ED symptoms found among Swedish elite athletes in the present study emphasises the need for implementing validated prevention strategies within the sporting environment, including programs for primary, secondary, and tertiary prevention programs [1, 88].

Maladaptive exercise, including exercise addiction, is frequent among individuals with ED symptoms and is associated with ED psychopathology and symptom severity, suicidal behaviour, psychiatric comorbidity, and poor treatment outcomes [89]. Since excessive exercise behaviour is often normalised within the sport environment, our results reinforce recent recommendations to screen for exercise addiction in all ED assessments [90]. Sports medicine providers therefore play a crucial role in early detection and a multidisciplinary approach, including a physician, a dietitian and a psychologist in the treatment team is essential [49].

The present findings underscore the importance of integrated screening for depressive symptoms to improve early detection, as DE behaviours are significantly associated with both current depressive symptoms and lifetime depressive disorders in competitive sport settings [25, 53]. Depression may therefore represent a key indication of elevated clinical risk among athletes with ED symptoms. Psychological inflexibility is closely linked to elevated depressive symptoms [83], and growing evidence suggests that inflexibility-related processes are strongly associated with ED psychopathology [85]. Together, these findings suggest that psychological inflexibility may constitute a shared vulnerability factor contributing to the co-occurrence of ED symptoms, depression and exercise addiction observed in our sample.

### Strengths and limitations

Major strengths of this study include the use of a two-step design incorporating both validated self-report instruments and gold standard clinical interviews, the inclusion of an age- and sex-matched comparison group, and the recruitment of athletes competing at national-team level across multiple sports. Several limitations need also be acknowledged. Recruitment procedures varied across sports federations, precluding calculation of a response rate and increasing the risk of selection bias. Self-reported anthropometric data may have introduced measurement error. The cross-sectional design prevents conclusions regarding causality or temporal relationships between ED symptoms and mental health variables. Finally, the relatively small number of male participants limited statistical power for sex-specific analyses.

## Conclusions

This study demonstrates that ED symptoms are common among Swedish elite athletes and are associated with depressive symptoms, exercise addiction, and psychological inflexibility. Although the prevalence of ED symptoms was higher in the comparison group, nearly one in five athletes screened positive for ED symptoms, highlighting the ongoing importance of ED prevention and early identification in elite sport. These findings support the implementation of systematic screening programmes, clear referral pathways, and multidisciplinary care models within sport organisations. Future research should examine longitudinal relationships between ED symptoms, psychological flexibility, and mental health outcomes to inform targeted prevention and intervention strategies.

## Data Availability

The data that support the findings of this study are available from the corresponding author (AKM), upon reasonable request.
